# To infinity and some glimpses of beyond

**DOI:** 10.1038/s41467-017-01502-7

**Published:** 2017-11-16

**Authors:** Panayotis G. Kevrekidis, Constantinos I. Siettos, Yannis G. Kevrekidis

**Affiliations:** 1 Department of Mathematics and Statistics, University of Massachusetts Amherst, Amherst, MA 01003-4515 USA; 2 School of Applied Mathematics and Physical Sciences, National Technical University of Athens, Athens, GR 15780 Greece; 3 Department of Chemical and Biological Engineering and Program in Applied and Computational Mathematics, Princeton University, Princeton, NJ 08544 USA; 4 Institute for Advanced Study, TUM, Munich, 85748 Germany; 5 Zuse Institute, Berlin, 14195 Germany

## Abstract

When mathematical and computational dynamic models reach infinity in finite time, extending analysis and numerics beyond it becomes a notorious challenge. We suggest how, upon suitable transformations, it may become possible to go beyond infinity with the solution becoming again well behaved and the computations continuing normally. In our Ordinary Differential Equation examples the crossing of infinity occurs instantaneously. For Partial Differential Equations, the crossing of infinity may persist for finite time, necessitating the introduction of buffer zones, within which an appropriate transformation is adaptively identified. Along the path of our analysis, we present a regularization process via complexification and explore its impact on the dynamics; we also discuss a set of compactification transformations and their intuitive implications. This methodology could be useful toward a systematic approach to bypassing infinity and thus going beyond it in a broader range of evolution equation models.

## Abstract (web summary)

Certain physical problems such as the rupture of a thin sheet can be difficult to solve as computations breakdown at the point of rupture. Here the authors propose a regularization approach to overcome this breakdown which could help dealing with mathematical models that have finite time singularities.

## Introduction

Mathematical models of physical, biological, as well as socio-economic phenomena and computations based on these models are often observed to approach infinity. This causes the computation to become exceedingly difficult, or to simply fail; returning from the neighborhood of infinity to more meaningful computational regimes is a notoriously hard task, often difficult to justify.

In the actual phenomena modeled, an approach toward infinity is often an indication of model breakdown. A convenient and ubiquitous illustrative example lies in studying self-similar solutions that collapse in finite time, a topic of widespread interest in both the mathematical and the physical literature. The contexts range from scaling^1,2^, to focusing in prototypical dispersive equations such as the Korteweg−de Vries (KdV) equation^3^ and most notably the nonlinear Schrödinger (NLS) equation^4–6^ on the one hand, and from droplets in thin films^7,8^ and flow in porous media^9,10^ to the roughening of crystal surfaces^11^ and integrate-and-fire neuronal models^12–14^ on the other. One may try to avoid collapse (e.g., by imposing space^15–17^ or time modulations^12,13,18,19^) or by identifying the higher order effects that preclude collapse in physical experiments^20^. One may alternatively explore what happens to the mathematical, computational (or even physical^21^) setting at, or past, the moment of collapse; see, e.g., the relevant chapter of ref. ^6^. In the context of semilinear parabolic equations, there have been extensive theoretical^22^ and computational^23,24^ efforts to understand the self-similar rescaling process and approach to the singularity formation.

Our motivation is simple and, while also physical in part (as in ref. ^21^, where the impact of collapse on optical filaments is sought), it is chiefly both mathematical and computational. As collapse is approached in time, computations naturally breakdown and so also do, in part, mathematical approaches; there are notable exceptions, e.g., efforts to explore beyond collapse, detailed in the book of ref. ^6^ for NLS, or in refs. ^9,10^ for the porous medium problem. This breakdown has motivated extensive efforts to refine computational meshes^25,26^ and avoid collapse at the numerical level (possibly transforming into a co-exploding frame, thus factoring out the self-similarity^5,27^ as will be discussed further below). Such numerical approaches do not, however, possess the ability to cross infinity, even in a simpler array of examples in which we know by construction, or via analytical arguments, that life past infinity persists (i.e., that the solution does not cease to exist and can be continued past a singular point). This is precisely our aim here: we will propose how to numerically go beyond infinity considering both ordinary (ODE) and partial differential equations (PDE), as if it was a regular, rather than a singular point. We construct and apply, on demand, a singular transformation that absorbs the singular nature of the dynamics, allowing the solution to re-emerge on the other side of infinity, where the dynamics becomes regular again. A complementary perspective that we explore in this regard is one of compactification transformations which place infinity on equal footing with the rest of the points. Our rescaling approach bears some similarities to—but also differences from—the well-known renormalization group approach in theoretical physics^28^.

In the present work, we use ODE and PDE models to showcase our ideas. In our ODE examples, including 1D and 2D versions of the integrate-and-fire neuronal models, an instantaneous encounter with infinity (crossing or otherwise, as will be discussed below) will be considered (and appropriately bypassed) in what follows. In the PDE examples the introduction of physical space leads to multiple possibilities; one is that collapse might only occur at a single physical point/moment in time, with no subsequent continuous crossing of infinity. This is the so-called transient blowup in the insightful summary of ref. ^29^ aiming at the classification (see the discussion of item (5) therein) of post-focusing regimes; we will return to it in our discussion. For PDEs, we will instead focus on the computationally intriguing case where, upon touching infinity at an initial point in space/time, the solution will start gradually crossing; in one spatial dimension this will generically result in two simultaneous crossings that emerge from the original encounter with infinity, and subsequently propagate apart in space/time. This poses computational challenges, as collapse persists in time (there needs to be a singular transformation in some portion(s) of the domain for entire time intervals), and it is also mobile; we thus proceed to adaptively follow the region(s) where the singular transformation is detected and accordingly performed as needed. It does not escape us that an additional possibility can be envisaged: finite spatial intervals of the solution (possibly multiple ones simultaneously) may become infinite, leading the regular part of the solution to be supported in compact regions, resembling the so-called compacton structures originally introduced in ref. ^30^. This is referred to as incomplete blowup in ref. ^29^. We also perform a complexification of the evolving variable(s) and illustrate how this may lead to a regularization of the real collapsing dynamics. This is, arguably, a topic of interest in its own merit; yet it connects naturally with the overall picture of approaching (and potentially crossing) infinity, and, as such, we will briefly discuss it here. It should be added that regularization as a subject has a time-honored history in its own right. A particularly informative summary of such efforts can be found in ref. ^31^.

## Results

### Ordinary differential equations

The standard textbook ODE for collapse in finite time (and its solution by direct integration) reads:

1 $$\dot x = x^2 \Rightarrow x(t) = \frac{1}{{t^ \star - t}}.$$

The collapse time t* = 1/*x*(0), is fully determined by the initial condition, and the textbook presentation usually stops here. A numerical solver would overflow close to (but before reaching) t*; yet we can bypass this infinity by appropriately transforming the dependent variable *x* near the singularity. Indeed, the good quantit*y y* ≡ 1/*x* ≡ *x* ^−1^, satisfies the good differential equation d*y*/d*t* = −1; this equation will help cross the infinity (for *x*) by crossing zero and smoothly emerging on the other side (for *y*). Once infinity is crossed, we can revert to integrating the initial (bad, but now tame again) equation for *x*.

The numerical protocol that we propose (see also Methods section) naturally circumvents problems associated with infinity in a broad class of ODEs that collapse self-similarly, as power laws of time (or, importantly, as we will see below in Methods section, also asymptotically self-similarly) and consists of the following steps:

1. Solve the bad ODE (e.g., Eq. (1)) for a while, continuously monitoring, during the integration, its growth toward collapse.
2. If/when the approach to collapse is detected, estimate its (asymptotically) self-similar rate (the exponent of the associated power law, e.g., −1 for $\dot x = x^2$) and use it to switch to a good equation for *y*, relying on the singular transformation *y* = 1/*x* with this exponent (and on continuity, to obtain appropriate initial data for this good equation). The relevant scaling law may not be straightforward to detect via the equations of motion, especially for self-similarity of the second kind^9^. Nevertheless, a numerical identification utilizing, e.g., the power-law relation between the numerical d*x*/d*t* and *x* could be well suited to such a case.
3. Run this good equation for *y* until 0 (or ∞ for the former, bad equation) is safely crossed, computationally observing for *x* an (asymptotically) self-similar return from infinity.
4. Finally, transform back to the bad equation (now tamed, as infinity has been crossed) and march it further forward in time.

**Fig. 1:**
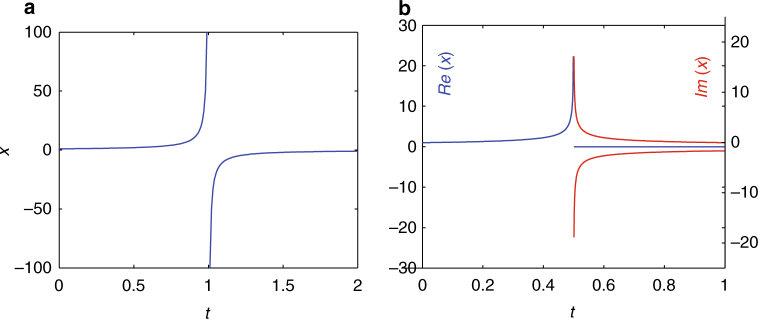
Infinity crossing in ODEs. The cases of **a** $\dot x = x^2$, *x*(0) = 1 leading to collapse at *t* ^⋆^ = 1 and, **b** of $\dot x = x^3$, *x*(0) = 1, leading to collapse at t* = 0.5; in this case *x*(*t*) becomes imaginary beyond t* (see also Methods section)

This protocol (see also Methods section) has been carried out in Fig. 1a, illustrating that the dynamics can cross infinity and computation can be continued for all time, provided that the self-similar approach to infinity is adaptively detected and the associated, and appropriately numerically estimated, singular transformation is used to cross it. In Fig. 1a, we solve $\dot x = x^2$ until the solution reaches *x*(*t*) = 100, followed by solving $\dot y = - 1$ beyond crossing 0 to *y*(*t*) = 0.01, and then returning to Eq. (1).

Note that the notion of compactification (Methods section) also allows the progression past infinity in time too, when now *y* crosses zero as time approaches positive infinity and then returns from negative infinity. To manifest the feature that infinity crossing should be thought of as being on equal footing with any other point on the rest of this orbit, we introduce such a notion of compactification^32^. Reshuffling the (hyperbolic form of the) solution of Eq. (1), we have

2 $$(t^ \star - t)x = 1 \Rightarrow \left( {\frac{{t^ \star - t + x}}{2}} \right)^2 - \left( {\frac{{t^ \star - t - x}}{2}} \right)^2 = 1.$$

Compactification through the variables *X* and *Y*

3 $$X = {\mathrm{cos}}(\theta ) = (t^ \star - t - x){\mathrm{/}}(t^ \star - t + x),$$

4 $$Y = {\mathrm{sin}}(\theta ) = 2{\mathrm{/}}(t^ \star - t + x).$$

converts this hyperbola to a circle; one can verify that indeed −1 ≤ *X* ≤ 1 and −1 ≤ *Y* ≤ 1 and also that *X* ^2^ + *Y* ^2^ = 1. This puts both relevant infinities

5 $$(t \to t^ \star ,x \to \pm \infty ) \Rightarrow (X \to - 1,Y \to 0^ \pm ),$$

6 $$(t \to \pm \infty ,x \to \pm 0) \Rightarrow (X \to 1,Y \to 0^ \mp ).$$

on equal footing with all other points of the orbit along the circle. The trajectory between the point (1, 0) (the infinity in *t*, the steady-state in *x*) and the point (−1, 0) (the infinity in *x*) can be thought of as reminiscent of a heteroclinic connection.

Such connections often arise in dynamical systems with symmetries (e.g., refs. ^33,34^). The compactification also suggests that, provided we utilize the right variables, i.e., the right quantities to observe the solution, (e.g., in the form of this circle) we should obtain a consistent, smooth picture (with consistent, smooth numerics). Indeed, $y(t) = 1{\mathrm{/}}x(t)( = t^ \star - t)$ is a transformation in itself singular, yet one which converts the bad exploding variable *x*(*t*) into a good variable *y*(*t*), satisfying d*y*/d*t* = −1 that merely smoothly crosses 0.

The collapse of $\dot x = x^3$ whose exact solution is $x(t) = 1{\mathrm{/}}\sqrt {t^ \star - t}$ is worth examining separately. The relevant singular transformation (Methods section) (here *y*(*t*) = 1/*x* ^2^) will take us to infinity in finite time, but, at first sight, will not cross *y*(*t*) becomes imaginary beyond t* (Fig. 1b). An appropriate compactificaton (Methods section) resolves the issue:

7 $$X = {\mathrm{cos}}(\theta ) = (t^ \star - t - x^2){\mathrm{/}}(t^ \star - t + x^2)$$

8 $$Y = {\mathrm{sin}}(\theta ) = 2/(t^ \star - t + x^2),$$

leading to perfectly regular dynamics on a circle, so that the singularity is again bypassed.

We can now try to extend/generalize these ideas to other collapse rates (i.e., arbitrary powers/exponents of self-similarity). For ODEs that asymptotically collapse self-similarly, $x(t)\sim 1{\mathrm{/}}(t^ \star - t)^a$, we can produce a useful compactification in the form

9 $$X = {\mathrm{cos}}(\theta ) = ((t^ \star - t)^a - x){\mathrm{/}}((t^ \star - t)^a + x)$$

10 $$Y = {\mathrm{sin}}(\theta ) = 2{\mathrm{/}}((t^ \star - t)^a + x).$$

In this form, the dynamics benignly travels along the circle. Relevant examples can straightforwardly be extended to, e.g., fractional powers although it is known from standard ODE analysis that issues of uniqueness may arise there that we do not delve into in the present work.

More generally then, the self-similarly collapsing ODE d*x*/d*t* = ±*x* ^*p*^ has the solution $\pm x^{1 - p}{\mathrm{/}}(1 - p) = t - t^ \star$ and its scaling in time follows $x(t)\sim (t^ \star - t)^{1/(1 - p)}$, with the collapse time once again determined by the initial data. Given a legacy code that integrates the ODE $\dot x = F(x)$, we monitor its growth approaching collapse (i.e., how *F*(*x*) scales as *x* ^*p*^, or more generally with ||*x*||). For vector cases, the analogous feature could be the monitoring of the norm dependence as ||*x*||^*p*^, although we will not explore such a case here. Upon detection of asymptotically self-similar collapse, at sufficiently large |*x*| (e.g., 10^2^ in the ODE of Fig. 1, or 10^4^ in the PDE example that will follow) we stop solving the bad ODE. We use instead the singular transformation *y* = *x* ^−*p*+1^ (for d*x*/d*t* = *f*(*x*), more generally, $y = {\int}_x^\infty 1{\mathrm{/}}f(s) {\mathrm d}s$ leads to d*y*/d*t* = −1) to solve the good ODE *y*(*t*) that crosses 0 rather than infinity. Then, a little beyond the collapse time (beyond infinity for *x*(*t*), beyond 0 for *y*(*t*)) we simply revert to the original, bad (yet no longer dangerous) ODE, with continuity furnishing the relevant matching conditions.

An illustration of asymptotically self-similar blowups, where different transformations are used to cross two different infinities (the finite time/infinite value and the infinite-time/finite value ones) is included in the Methods section.

Examining such infinity crossings as regular, rather than singular points begs an explanation for the mechanism of exiting the real axis along +∞ and then re-emerging on the other side at −∞ (for d*x*/d*t* = *x* ^2^) or arguably more remarkably from +*i*∞ back toward the origin in the example involving *x* ^3^). In the latter case there is an obvious ambiguity: the solution might just as well be chosen to re-emerge from −*i*∞: one can formally, past the collapse, accept $x(t) = \sqrt { - 1{\mathrm{/}}(t - t^ \star )} = i{\mathrm{/}}\sqrt {t - t^ \star }$ for *t* > t* or, alternatively, $x(t) = \sqrt {1{\mathrm{/}}( - (t - t^ \star ))} = - i{\mathrm{/}}\sqrt {t - t^ \star }$. This is perhaps a prototypical (and tangible) example of the phase loss feature argued in refs. ^6,21^. As a way of shedding further light into these features, we provide a complexified version of d*x*/d*t* = *x* ^2^ and of d*x*/d*t* = *x* ^3^ in the Methods section, which provides insights on these crossings and on the time that it takes for them to occur (also explicitly computed in the Methods section).

Moreover in the Methods section, to show the applicability of the proposed methodology to biologically/physically inspired examples, we illustrate one such from computational neuroscience, namely the celebrated quadratic integrate-and-fire-model (see also in the Methods section) reading^12^: d*v*/d*t* = *I* + *v* ^2^. To further cement the generality of the method beyond single degree of freedom examples, we also consider a 2 species ODE, namely the integrate-and-fire quadratic and quartic models exhibiting fast and slow dynamics, and illustrate that the ideas can be naturally generalized in such a setting. In particular, these models may be represented by the following general form:

11 $$\frac{{{\mathrm{d}}v}}{{{\mathrm{d}}t}} = I + F(v) - u,\quad \frac{{{\mathrm{d}}u}}{{{\mathrm{d}}t}} = bv - u.$$

It has been shown^13^ that in the quadratic model corresponding to *F*(*v*) = *v* ^2^, the adaptation variable, *u*, blows up at the same time with the membrane potential, *v*, while in the quartic model corresponding to *F*(*v*) = *v* ^4^ + 2*v*, the adaptation variable, *u*, remains bounded when *v* blows up^14^.

### A partial differential equation case

**Fig. 2:**
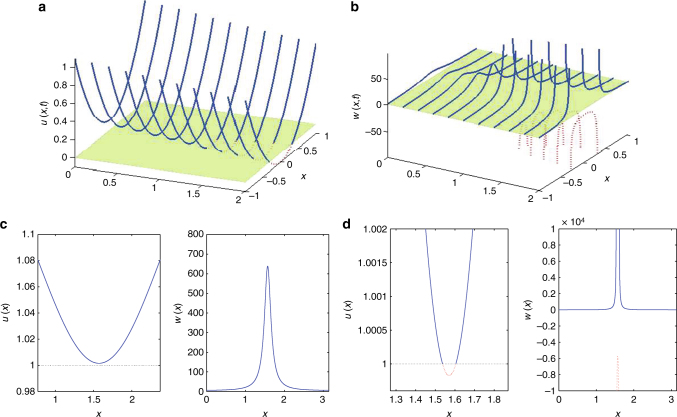
A geometry for crossing infinity for PDEs. **a**, **b** An idealized parabola *u*(*x*, *t*) **a** shifting at constant speed without change of shape, crossing the 0 level, **b** *w*(*x*, *t*) = 1/(*u*(*x*, *t*) − 0). **c**, **d** Solutions *u*(*x*, *t*) of Eq. (12) around their crossing of the 1 level, and corresponding *w*(*x*, *t*) = 1/(*u*(*x*, *t*) − 1) (solutions of Eq. (13)). **c** Just before crossing, and **d** just after crossing infinity

We now turn to a PDE example, illustrating one of the ways that space dependence modifies the crossing of infinity. Motivated by d*x*/d*t* = ±*x* ^2^, where *x* ^−1^ crosses infinity at a single moment in time, we study the case where infinity is first reached in finite time, and then crossed continuously in time, but (in one spatial dimension) at isolated points in space. The geometry involved is illustrated in Fig. 2a, showing a graph of the function *u*(*x*, *t*) = *x* ^2^ + (0.1 − 0.1*t*), a parabola shifting its values downward, at constant speed, but without change of shape, crossing the 0 level. Initially it is everywhere positive; the tip reaches the zero-level-set at t* = 1 and then crosses it. The function *w*(*x*, *t*) = 1/*u*(*x*, *t*) is shown Fig. 2b: it reaches infinity at t* = 1 and subsequently crosses at two points that move apart as dictated by the motion of the parabola. In Fig. 2c, d are shown solutions *u*(*x*, *t*) of Eq. (12) around their crossing of the 1 level, and the corresponding *w*(*x*, *t*) = 1/(*u*(*x*, *t*) − 1) (solutions of Eq. (13)). Figure 2c corresponds to the solutions just before crossing, and Fig. 2d to solutions just after crossing infinity.

We can then agree that the waveform returns from minus infinity between these two crossing points as the definition of *w*(*x*, *t*) formally suggests. Extension to higher-dimensional geometries (e.g., a paraboloid initially touching a plane at a point and then crossing it along a closed curve, such as a circle, that opens up starting at the initial point in two spatial dimensions) can also naturally be envisaged. The key observation is that the evolution of *w*(*x*, *t*) actually involves a (potentially asymptotically) self-similar collapse near the crossing of infinity.

This suggests that, upon detection—on the fly—of such an asymptotically self-similar collapse and estimation of the associated exponents (see below) for a bad *w*(*x*, *t*) PDE, a search for a good observable *u*(*x*, *t*) be performed. Then,a conceptual and computational program analogous to that of the previous Section on ODEs may be carried through to obtain, and work with, a good PDE in the vicinity of the collapse point.

The simple, linear equation

12 $$u_t = u_{xx} - u,$$

provides an engineered, yet transparent and analytically tractable illustration of the relevant ideas and hence will be used as our workhorse in what follows. Generic initial data in this well-posed, linear model decay and concurrently spread, asymptoting to *u*(*x*, *t*) = 0 at long times. We select an arbitrary level set u* = *r* > 0 and a modified variable *w* ≡ 1/(*u*(*x*,*t*) − *r*) to study level set crossings; for initial conditions everywhere above *r*, and on its way to zero, *u*(*x*, *t*) will cross the level set *r* so that *w*(*x*, *t*) will cross the level set at infinity.

The bad PDE for *w*(*x*,*t*) reads

13 $$w_t = w_{xx} - \frac{2}{w}w_x^2 + w + rw^2.$$

An auxiliary tool for the analysis of (asymptotically) self-similar collapse in such equations is the so-called MN-dynamics^27,35^; a dynamic renormalization scheme rescaling space^36^, time and the amplitude of the solution so that the self-similar solution becomes a steady-state in the co-exploding frame, i.e., the frame factoring out the symmetry/invariance associated with the (potentially asymptotic) self-similarity. This formulation is presented as a separate, detailed section in Methods for completeness. In that section, both the general case, and the special example of Eq. (13) are treated. From this formulation we can infer that $w\sim 1{\mathrm{/}}(t^* - t)$, which, in turn, suggests the choice of a good variable used below.

In our illustrative example we use Neumann Boundary Conditions (BC) in [0, *π*] and initial conditions *u*(*x*, 0) = *a* cos(2*x*) + *c* (here *a* = 0.4, *c* = 1.5), so that the solution *u*(*x*, *t*) of Eq. (12) reads:

14 $$u(t) = 0.4\,{\mathrm{exp}}( - 5t){\mathrm{cos}}(2x) + 1.5\,{\mathrm{exp}}( - t),$$

and we choose *r* = 1. We do not, however, pre-assume such knowledge of *u*(*x*, *t*) since the equation we have to solve is the bad (focusing) *w*(*x*, *t*) equation, i.e., Eq. (13); our MN framework applied to the focusing of the *w*(*x*, *t*) evolution then suggests that a good observable is *v*(*x*, *t*) ≡ *w*(*x*, *t*)^−1^, a variable that will simply be crossing 0 and thus the good PDE would simply be

15 $$v_t = v_{xx} - v - r.$$

Recall that our goal is to seamlessly carry out the computation without our numerical code ever realizing that (some part of) the solution is becoming indefinitely large. To achieve this, as the bad PDE solution grows toward infinity, it is adaptively tested, with a user-defined threshold for (local, asymptotic) self-similarity, i.e., for growth according to a (potentially approximate) power law. When this is numerically confirmed, a suitable power law transformation is devised with the numerically estimated similarity exponent; in the above example the detected exponent is −1 and so the transformation is *v* = *w* ^−1^. The easiest way to realize the right observable in this case is to consider uniform initial conditions—then the PDE reduces to an ODE that asymptotically explodes as the $\dot w = w^2$, suggesting the *v* = *w* ^−1^ change of observables.

**Fig. 3:**
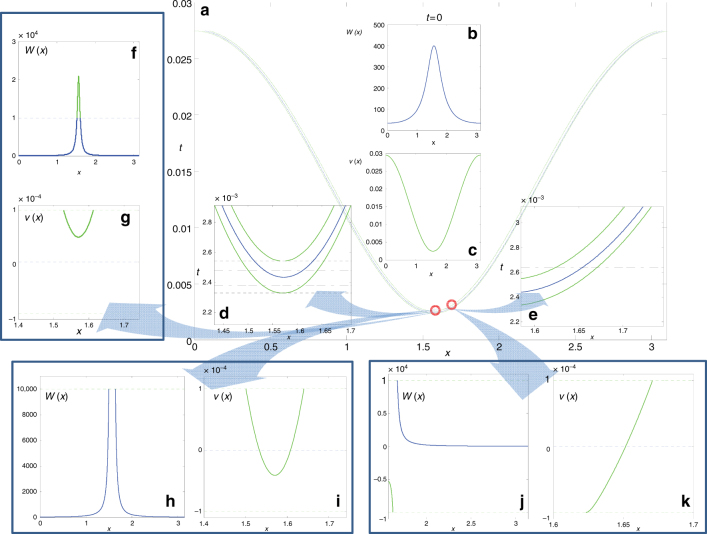
Schematic of the computation for the PDE paradigm. The main background **a** shows the locus (in blue) of the points in space-time where *w* becomes infinite. The two accompanying (green) solid curves define the bands within which we solve the good equation for ν = 1/*w*. The central insets in **a**, i.e., **b**, **c** show the initial bad equation profile *w*(*x*) **b** and the good profile ν(*x*) **c** at *t* = 0. Insets **d**, **e** zoom at specific points. Insets **f**, **g** take place after we have crossed our selected high level (here 10^4^) for *w* defining the buffer region boundary, but have not yet crossed infinity; both the bad (*w*) **f** and good (ν) **g** solutions are shown. Insets **h**, **i** take place after *w* crossed infinity, but has not (in absolute values) receded below the high level defining the buffer regions. Again both the bad (*w*) **h** and the good (ν) **i** solutions are shown. Insets **j**, **k** show the solution of the good equation ν **k** (inside the band) and of the bad equation *w* **j** (outside the band) after the singularity point has started propagating to the right (and left)

Importantly, the transformation has to be performed—and the good solution sought—over an entire spatial interval(s) surrounding the approaching singular point(s). This suggests the following procedure, illustrated schematically in Fig. 3:

1. Upon detection of approach to infinity as the tip of the collapsing waveform grows beyond a sufficiently large value at a given point or points inside the computational domain, we split the domain in 3 regions: two regular ones to the left and to the right of the growing tip, where the original bad equation for *w* is being solved; and a new singular one, in the middle, where instead of solving the equation for *w*, now the equation for its singularly transformed variant, the good equation for *v* = *w* ^−1^ is solved instead. The latter transformation is selected to comply with either the self-similar analysis on the theoretical side, or the identified power law of amplitude growth on the numerical side. These equations are linked by continuity of the (transformed) observables at the domain boundaries, and standard domain decomposition numerical techniques are used^37^. The good equation simply crosses zero rather than crossing infinity, as in the ODE case.
2. Once zero is crossed, the initial single crossing point in the family of case examples under consideration (in one spatial dimension) opens up into two infinity crossings (one can visualize two waves that propagate in opposite directions, one to the left and one to the right)—two zero-level-set crossings for the good equation. These crossings are quantified, for our example, in Fig. 3. They are bordered by the computed locations of a high enough absolute level (here 10^4^) for asymptotic self-similarity.
3. To deal with the two new crossings computationally over time, the central region is subsequently split into 3 regions. The two outer ones are our singular buffers, surrounding, and in some sense masking the infinity crossings to the left and to the right. But now they are separated by another, inner regular interval, where we can again solve the original bad equation since in here it is again sufficiently far from infinity. Thus, post collapse, we partition the domain into five regions, three regular ones—the two outer ones, and the innermost, for the bad equation—and then two singular buffers for the transformed good equation, one around the left zero crossing and one around the right zero crossing of *v*, that correspond to the two infinity crossings of the bad equation for *w*.

A nontrivial aspect of the computation is the gluing between the regular regions and the singular buffers. Our numerical scheme here is a simple one following^37^: for a finite difference discretization in space, (a) an explicit forward Euler time step is performed at the interface points, which provides the interior boundary conditions for the next time step (the next computational era), while (b) an implicit Euler time step is adopted to solve the good and bad PDE within the three (or the five) domains. The scheme can be modified to allow for different space and time steps in the different domains till the next computational era, when the new interface points will be detected, the new interior BCs will be computed, and the good and bad PDE in each domain will be solved. To recap the essence of the algorithm, by solving the good equation inside the buffer regions (and following the motion of the buffers on the fly), we ensure that the numerical simulation is never plagued by the indeterminacy associated with approaching/touching/crossing infinity.

The panels of Fig. 3 show representative instances just before and just after the initial encounter of the *w*(*x*, *t*) profile with infinity in both its good *v*(*x*, *t*) ≡ (*u*(*x*, *t*) − *r*) and its bad *w*(*x*, *t*) incarnations, in the spirit of Fig. 2a, b.

The approach to infinity for *w*(*x*, *t*) is indeed asymptotically self-similar, as explained in the Methods section. As we approach the event, an inverted bell-shaped profile comes close to, touches, and then starts crossing through *r* = 1 in the variable *u*, or crossing through 0 in the variable *v* ≡ *u* − *r*, or equivalently crossing through ∞ in the variable *w*. Using the good variable *v* inside appropriate buffer regions, and the bad variable *w* outside of these, as explained in the Methods section below, we are thus able to continue the computation for all time, avoiding the singularity of the bad PDE.

We now explore two more notions that were also examined in the ODE context. The first is compactification: self-similar PDE dynamics, although crossing through infinity, can simply be compactified as evolving over a circle or -better- on a sphere. We perform this step for a general 1D PDE (self-similar) solution of the form –assuming independence from *τ* (see the MN formulation in the Methods section):

16 $$u(x,t) = \frac{1}{{(t^ \star - t)^r}}f(\xi ),$$

where *ξ* is a self-similar variable, e.g., $\xi = x{\mathrm{/}}(t^ \star - t)^q$. If the max(*f*) >1, we can define $\tilde f = f{\mathrm{/max}}(f)$ (as well as rescale *u* by the same factor) and subsequently drop the tilde in Eq. (16) ensuring that $\left| f \right|$ ≤ 1. We can then rewrite Eq. (16) as:

17 $$(t^ \star - t)^ru = f \Rightarrow \left( {(t^ \star - t)^r + u} \right)^2 - \left( {(t^ \star - t)^r - u} \right)^2 = 4f(\xi ).$$

Then, upon suitable definition of the variables, we can have $X = \left( {(t^ \star - t)^r - u} \right){\mathrm{/}}\left( {(t^ \star - t)^r + u} \right)$ and $Y = 2\sqrt {\left| {f(\xi )} \right|} {\mathrm{/}}\left( {(t^ \star - t)^r + u} \right)$, in which case *X* ^2^ + *Y* ^2^ = 1. In these (or similar) variables, at every moment in time the trajectory can be thought of as compactified along a circle. However, as the circle itself represents an invariant shape, in this representation we cannot straightforwardly visualize the trajectory’s dynamics; for this reason, we next compactify the dynamics on a sphere. We define *g* ^2^ = 1 − *f* ^2^ and we can then write using the above variables (*gX*)^2^ + (*gY*)^2^ = *g* ^2^ = 1 − *f* ^2^, which can be reshuffled to read:

18 $$\left( {g\frac{{(t^ \star - t)^r - u}}{{(t^ \star - t)^r + u}}} \right)^2 + \left( {g\frac{{2\sqrt {\left| f \right|} }}{{(t^ \star - t)^r + u}}} \right)^2 + f^2 = 1.$$

Choosing the three terms of the left hand side of Eq. (18) as $(X{\prime},Y{\prime},Z{\prime}) = (g((t^ \star - t)^r - u)/((t^ \star - t)^r + u),g\left( {2\sqrt {\left| f \right|} } \right){\mathrm{/}}((t^ \star - t)^r + u), f)$ we observe that the dynamics can be seen as evolving along the surface of a sphere. This compactification once again underscores the possibility to think of infinity as a regular circle (rather than point, as is the case for ODEs), a level set that is crossed by the PDE solution evolving along the surface of the sphere. Furthermore, as in the case of ODEs, we also examine the complexified version of Eq. (13) in the Methods section.

## Discussion

We attempted to address here a few prototypical cases of a spectrum of problems arising in both ordinary and partial differential equations, so as to deal with the emergence of infinities during the evolution of the relevant models. In a number of cases the model at hand will become physically inaccurate, and will need to be suitably modified as these singular points are approached; if not, our questions may be relevant for the physical realm. In any event, the questions are of particular relevance toward the mathematical analysis and numerical computation of the models at hand. In that light, we argued that it is possible in our context to perform singular transformations on demand, that may sidestep—through the help of a suitable good equation—the computational difficulties associated with infinities, rendering them tantamount to the crossing of a regular point such as zero. For ordinary differential equations, once the crossing has transpired, one can safely return to the original bad equation and continue the dynamics from there (until possibly a new infinity is approached).

In the case of partial differential equations, the scenario at hand is more complex. There, the solution is distributed in space, and hence we assume and have analyzed the setting where a (generically assumed to be parabolic; see the relevant discussion in the Methods section) tip of a waveform approaches infinity. We have discussed in detail a scenario of initially touching infinity and then crossing it. Suitable computational buffers need then to be devised, where the detected singular transformation allows us to locally re-interpret (for computational purposes) the crossing of infinity as the crossing (in a transformed space) of a regular point, such as zero. These buffers need to be in constant and consistent communication, through appropriate continuity conditions, with the rest of the computational domain (the rest of the world). Typically, the buffers are defined by the location at which the solution takes on a sufficiently large (absolute) value—say 10^4^ to the left and right of the growing tip in the pre-crossing regime, or, say, 10^4^ till −10^4^ on the left, and −10^4^ till 10^4^ on the right in the post-crossing regime.

Our computational findings were complemented by a compactification approach, supporting the argument that infinity can be addressed in the same way as a regular point or a regular level set along the orbit. At the same time, a complexification of the model –discussed more at length in the Methods section– is observed to provide a regularization of the original real dynamics, avoiding the collapse of the latter and offering insight on how collapsing orbits can be envisioned as limiting scenarios of nonlinear dynamical systems within the complex plane.

## Methods

### Complexification of ordinary differential equations

The complexified version $\dot z = z^2$ (*z* = *x* + *iy*) leads to the two-dimensional dynamical system:

19 $$\dot x = x^2 - y^2,\quad \dot y = 2xy.$$

**Fig. 4:**
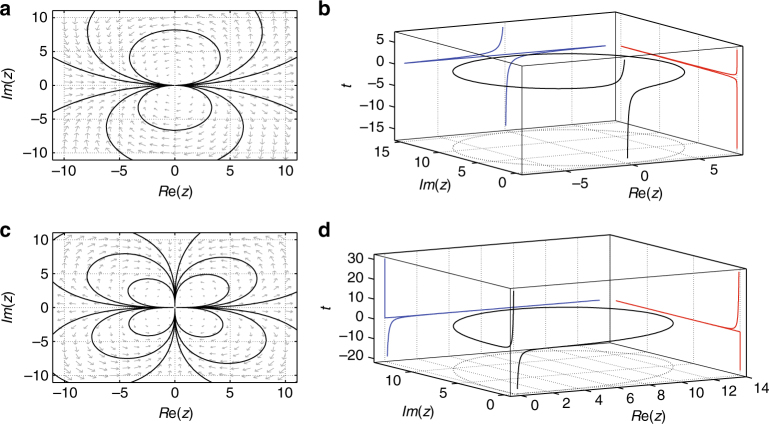
Complexified variants of ODE models. **a**, **b** The complex dynamics of $\dot z = z^2$ as represented by ODEs of Eq. (19). Phase plane analysis **a** and sample trajectory **b**. **a** Shows that the orbits close (and are, in fact, circles as shown in the text). **b** Illustrates the circular nature of the projection in the bottom *x* − *y* plane, as well as the *x* − *t* and *y* − *t* plane projections while following the *x* − *y* − *t* composite trajectory. **c**, **d** The complex dynamics of the two-degree-of-freedom system with $\dot z = z^3$. An example from the first quadrant of **c** is illustrated in more detail in the **d**, exhibiting how collapse is avoided in this case

The real axis is an invariant subspace, retrieving our real results; yet complexification endows the dynamics with an intriguing capability: as Fig. 4a, b illustrates through the (*x*, *y*) phase plane, collapse is avoided in the presence of a minuscule imaginary part. Large elliptical-looking trajectories are traced on the phase plane, eventually returning to the neighborhood of the sole fixed point of (0, 0)—which in the real case one would characterize as semi-stable. The system of Eq. (19) can be tackled in closed form since the ODE $\dot z = z^2$ yields 1/*z* = −*t* + 1/*z*(0). For *z* = *x* + *iy* (*z*(0) = *x*_0_ + *iy*_0_) we obtain the explicit orbit formula

20 $$x(t) = \frac{{x_0\left( {x_0^2 + y_0^2} \right) - t\left( {x_0^2 + y_0^2} \right)^2}}{{\left( {x_0 - t\left( {x_0^2 + y_0^2} \right)} \right)^2 + y_0^2}},$$

21 $$y(t) = y_0\frac{{x_0^2 + y_0^2}}{{\left( {x_0 - t\left( {x_0^2 + y_0^2} \right)} \right)^2 + y_0^2}}.$$

Eliminating time by dividing the two ODEs within Eq. (19) directly yields an ODE for *y* = *y*(*x*) (rather than the parametric forms of Eqs. (20), (21)). From this ODE, one can obtain that the quantity

22 $$E = \frac{{y^2 + x^2}}{y} = \frac{{y_0^2 + x_0^2}}{{y_0}},$$

is an invariant of the phase plane dynamics, and thus the latter can be written as *x* ^2^ + (*y* − *R*)^2^ = *R* ^2^, where $R^2 = \left( {x_0^2 + y_0^2} \right)^2{\mathrm{/}}\left( {4y_0^2} \right)$. That is, the trajectory evolves along circles of radius *R* in the upper (resp. lower) half plane if *y*_0_ > 0 (resp. *y*_0_ < 0.) Approaching the axis with *y*_0_ → 0, the curvature of these circles tends to 0 and their radius to ∞ (retrieving the real dynamics as a special case). Figure 4 through its planar projections illustrates not only the radial projection of the dynamics in the *x* − *y* plane, but the *x* − *t* and *y* − *t* dependencies.

Starting with a minuscule imaginary part the real dynamics tends to infinity; yet when the real part gets sufficiently large (somewhat in the spirit of our computations above), the imaginary part takes over, grows rapidly, and chaperons the real part to the negative side. Once the real part reaches the opposite (absolutely equal) negative value, the imaginary part rapidly shrinks and the formerly bad, yet now benign real equation takes over again.

We point out here that there is also a canonical way to generalize the compactification of this complex picture to the Riemann sphere through the inverse stereographic projection

23 $$X = \frac{{2x}}{{x^2 + y^2 + 1}},$$

24 $$Y = \frac{{2y}}{{x^2 + y^2 + 1}},$$

25 $$Z = \frac{{x^2 + y^2 - 1}}{{x^2 + y^2 + 1}}.$$

Now the real dynamics become a great geodesic circle, while all other complex plane curves become regular circles on the surface of the sphere. Under this transformation all points with *x* ^2^ + *y* ^2^ → ∞ are identified with (0, 0, 1), rationalizing the vanishing time needed to move from one to the other.

For the cubic case $\dot z = z^3$ the two-dimensional dynamical system becomes

26 $$\dot x = x^3 - 3xy^2,$$

27 $$\dot y = 3x^2y - y^3.$$

The corresponding phase portrait is shown in Fig. 4c, while a typical trajectory is shown in figure Fig. 4d. Instead of one leaf in the upper half plane there are now two leaves, one in each quadrant, see Fig. 4c; this suggests a natural generalization to *n* − 1 leaves in each half plane in the case of $\dot z = z^n$. It does not escape us here that a particularly intriguing case in its own right is whe*n n* is rational and perhaps even more so when it is irrational. However, we will restrict our considerations to the simpler integer cases herein, deferring the rest to future work. In the cubic case there is collapse for both positive and for negative initial data, and reentry along either the positive (resp. the negative) imaginary infinity (i.e., from +*i*∞, resp.−*i*∞) could be chosen (in analogy to the arbitrariness in the phase factor).

However, for even infinitesimally small imaginary data, the symmetry is broken, and unique trajectories are selected along each quadrant. A small real part (accompanied by a small imaginary part) as Fig. 4d grows until eventually (when sufficiently large) the imaginary part takes over. The real part then decays rapidly to 0, while the imaginary decays slowly, closing the orbit in the quadrant of the initial conditions; this is again a natural extension of the limiting case of purely real initial data. This complex formulation also allows the quantification (in a vein similar to ref. ^38,39^) of how long it takes for initial data, say, on the real axis, to emerge on the imaginary axis. This time (see below in the Methods section) tends to 0 for the transitions between +∞ and +*i*∞ for $\dot z = z^3$ (or from +∞ to −∞ in $\dot z = z^2$).

### Complexification of partial differential equations

As in the ODE case, we discuss the possibility of complexifying the model in order to understand, as a limiting case, how infinity is crossed for purely real initial data, while it may be avoided (regularized) upon initialization with complex initial data. Using the complex decomposition for *w*(*x*) = *a*(*x*) + *ib*(*x*) in Eq. (13), one can obtain the pair of real and rather elaborate looking equations:

28 $$a_t = a_{xx} - 2a\frac{{a_x^2 - b_x^2}}{{a^2 + b^2}} - 4b\frac{{a_xb_x}}{{a^2 + b^2}} + a + r(a^2 - b^2),$$

29 $$b_t = b_{xx} + 2b\frac{{a_x^2 - b_x^2}}{{a^2 + b^2}} - 4a\frac{{a_xb_x}}{{a^2 + b^2}} + b + 2rab.$$

**Fig. 5:**
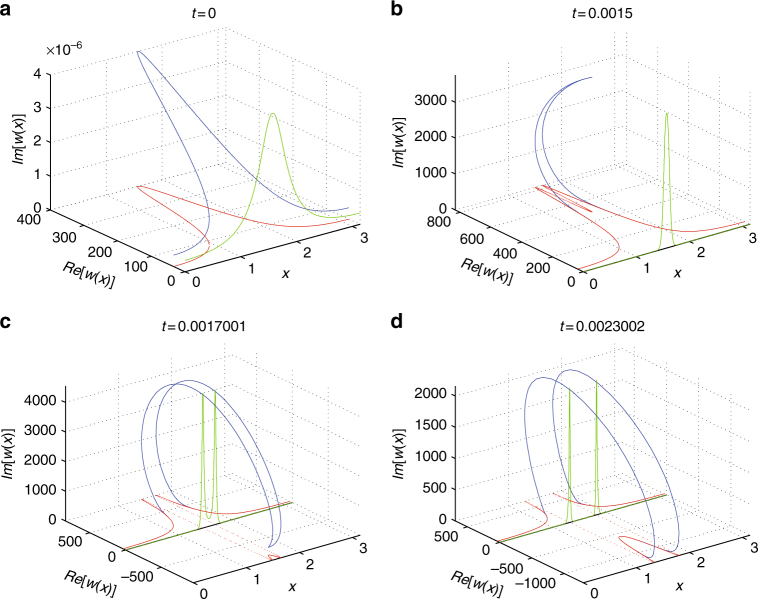
Simulation of the complexified form of the PDE model. The solution of the complex equation associated with the real and imaginary parts of Eqs. (28), (29) at various time instances **a** *t* = 0, just before the collapse time **b**, where the complex solution starts going back on itself rather than collapse (as it would for purely real initial data); **c**, **d** snapshots of the solution re-emerging on the other side (the negative real axis end of the complex plane). Notice the visual similarity with the images in Fig. 2

We expect that the presence of an imaginary part in the initial data may avoid collapse in analogy with Fig. 4. Given the quadratic nature of the nonlinearity, the quadratic ODE example is especially relevant; we expect here to observe something similar but in a PDE form, having space as an additional variable, over which the profile is distributed (around the crossing tip). Again, the tractability of our example allows us, via the solution of Eq. (15), to perform the relevant calculation analytically since at the level of the equation for *v* the complex model can be fully solved. Then, assuming *v* = *c* + *id*, the variable *w* = *a* + *ib* = 1/*v* = 1/(*c* + *id*) leads to *a* = *c*/(*c* ^2^ + *d* ^2^) and *b* =−*d*/(*c* ^2^ + *d* ^2^), and obtaining (*c*, *d*) explicitly, the same can be done for (*a*, *b*). This program is carried out in Fig. 5. We reconstruct analytically the spatial profile of the real and imaginary parts of *w* at different moments in time.

While the profile tends toward collapse in the real part of the variable (and would go all the way to collapse) the imaginary part, in analogy to the dynamics of Fig. 4, but in a distributed sense around the tip, eventually takes over. As it does so, it forces the solution filament to turn around on itself in a spatially distributed generalization of the ODE of Fig. 4. Finally, the solution appears to re-emerge from the other side, practically extinguishing its imaginary part, and having avoided the crossing of infinity. This illustrates how complexification, even in the case of the PDE, results in the avoidance of collapse and the regularization of the model; the collapsing real case is a special limit case of the more general complex one.

### Parabola self-similar crossing

In the 1D case, starting from the assumption of a self-similar solution approaching infinity, we can prescribe a generic unimodal profile of the form:

30 $$u\sim \frac{1}{{(t^ \star - t)^a}}f\left( {\frac{{x - x_0}}{{(t^ \star - t)^b}}} \right),$$

where t* is the collapse time and *x*_0_ is the point around which the blowup solution is centered.

Then, around *x* = *x*_0_ (and for $t \ne t^ \star$), we can use a Taylor expansion locally in the form:

31 $$u\sim \frac{1}{{(t^ \star - t)^a}}\left[ {f(0) + f{\prime}(0)\frac{{x - x_0}}{{(t^ \star - t)^b}} + \frac{{f{\prime\prime}(0)}}{2}\frac{{(x - x_0)^2}}{{(t^ \star - t)^{2b}}}} \right].$$

Combining the powers and bringing the dominant power to the left, we obtain that the field *v*, defined as

32 $$v \equiv u(t^ \star - t)^{a + 2b}\sim \left[ {f(0)(t^ \star - t)^{2b} + \frac{{f{\prime\prime}(0)}}{2}(x - x_0)^2} \right],$$

behaves like a regular field which crosses *v* = 0 at *x* = *x*_0_, when $t = t^ \star$. So, its dynamics should be that of a rising parabola, cutting through 0 at the critical time. In Eq. (32), we also used the fact that *x* = *x*_0_ was an extremum (having in mind in particular a maximum) of the profile of the solution (hence *f*′(0) = 0).

### MN-dynamics

As an auxiliary tool in our analysis, we will outline here and utilize the so-called MN-dynamics (see ref. ^35^), i.e., the self-similar dynamical evolution of a PDE which is collapsing toward a dynamical formation of a singularity. This approach has been used in porous medium type equations, as well as in dispersive (and conservative) NLS equations (see ref. ^20^ in the main text) and is broadly applicable to problems with self-similar growth (or decay). To illustrate it in a general form, we consider an evolutionary PDE of the form:

33 $$u_t = {\cal L}[\partial _\xi ]u + {\cal N}[u],$$

By ${\cal L}$ here we designate the operator involving derivatives (which we will consider to be a linear operator in what follows, although more generally products of powers of derivatives can also be tackled), while by ${\cal N}$ we designate the local nonlinearity bearing operator again here having in mind some power of *u*.

Using the ansatz

34 $$u = A(\tau )f(\xi ,\tau );\quad \xi = \frac{x}{{L(\tau )}},\quad \tau = \tau (t),$$

we introduce a new scaled system of coordinates, intended to be suitably adjusted to the self-similar variation of the PDE solution. *ξ* is a rescaled spatial variable (taking into consideration the shrinkage or growth of the width), while *τ* is a rescaled time variable, not a priori tuned, but which will be adjusted so that in this co-exploding frame, we factor out the self-similarity, in the same way in which when going to the co-traveling frame, we factor out translation. This way, the self-similar solution resulting in this dynamical frame will appear to be steady. Direct substitution of Eq. (34) inside of Eq. (33) yields:

35 $$\left[ {A_\tau f + Af_\tau - A\xi f_\xi \frac{{L_\tau }}{L}} \right]\tau _t = {\cal L}[\partial _\xi ]f\frac{A}{{L^a}} + A^s{\cal N}[f],$$

where *a* and *s* are powers tailored to the particular problem (linear and nonlinear operators) of interest. In order to match the scalings of the two terms of the right hand side of Eq. (35), as is required for self-similarity, we demand that:

36 $$\frac{1}{{L^a}} = A^{s - 1} \Rightarrow A\sim L^{ - \frac{a}{{s - 1}}} \Rightarrow G \equiv \frac{{A_\tau }}{A} = - \frac{a}{{s - 1}}\frac{{L_\tau }}{L}.$$

Thus, the model can now be rewritten as:

37 $$\left[ {G\left( {f + \frac{{s - 1}}{a}\xi f_\xi } \right) + f_\tau } \right]\tau _t = A^{s - 1}\left( {{\cal L}[\partial _\xi ]f + {\cal N}[f]} \right).$$

Demanding then that the time transformation be such that there is evolution toward a stationary state in this co-exploding frame, we remove any explicit time dependence by necessitating that $\tau _t = A^{s - 1}\sim L^{ - a}$. Then, the stationary state in this frame will satisfy:

38 $$G\left( {f + \frac{{s - 1}}{a}\xi f_\xi } \right) = {\cal L}[\partial _\xi ]f + {\cal N}[f].$$

It should be mentioned here that this analysis already provides an explicit estimate for the growth/shrinkage of amplitude and width over time, given that we assume that *A*_*τ*_/*A* = *G* = const. In particular, *A*_*t*_ = *A*_*τ*_ *τ*_*t*_ = *A*_*τ*_ *A* ^*s*−1^ = *GA* ^*s*^, which in accordance to the considerations of the previous section leads to the evolution of $A\sim (t^ \star - t)^{1/( - s + 1)}$. A similar analysis can be performed for *L* such that *L*_*t*_ = *L*_*τ*_ *τ*_*t*_ = *L*_*τ*_ *L* ^−*a*^ = −*G*[(*s* − 1)/*a*]*L* ^1−*a*^, leading to $L\sim (t^ \star - t)^{\frac{1}{a}}$. As a result of this analysis, our pulse-like entity touching (and potentially crossing) infinity will do so in a self-similar manner.

For the specific example of Eq. (13), using *w* = *Af*(*ξ*, *τ*), we obtain that

39 $${\cal L}[\partial _\xi ]w = \frac{A}{{L^2}}\left( {w_{\xi \xi } - \frac{2}{w}w_\xi ^2} \right);\quad {\cal N}[w] = Aw + A^2w^2.$$

It is then evident that the dynamics is not directly self-similar (due to the different scaling of the two terms within ${\cal N}$), but only asymptotically self-similar. When *w* (and *A*) is small, the exponential growth associated with the linear term is dominant. However, as the amplitude increases, eventually the quadratic term takes over, leaving the linear term as one of ever-decreasing-significance offending to the exact self-similar evolution. When the linear term becomes negligible, the self-similar evolution requires that *A*/*L* ^2^ = *A* ^2^, providing the scaling of $A\sim 1{\mathrm{/}}L^2$, i.e., in this case *s* = 2 and *a* = 2 for the general formulation above. From there, all the scalings associated with self-similarity can be directly deduced as explained previously.

### The linear ODE case

The mapping of the dynamics onto a circle can also be performed for the case of the simple exponential (rather than the power law self-similar, finite time collapse) arising from the simple linear ODE of the form:

40 $$\dot x = \pm x,$$

with the standard solution (assuming without loss of generality, positive initial data)

41 $$x(t) = e^{ \pm \left( {t - t^ \star } \right)}.$$

Here the dynamics can be written in hyperbolic form as

42 $$\left( {\frac{{e^{ \mp \left( {t - t^ \star } \right)} + x}}{2}} \right)^2 - \left( {\frac{{e^{ \mp \left( {t - t^ \star } \right)} - x}}{2}} \right)^2 = 1,$$

and the variables

43 $$X = {\mathrm{cos}}(\theta ) = \frac{{e^{ \pm (t^ \star - t)} - x}}{{e^{ \pm (t^ \star - t)} + x}},$$

44 $$Y = {\mathrm{sin}}(\theta ) = \frac{2}{{e^{ \pm (t^ \star - t)} + x}},$$

can be defined so that *X* ^2^ + *Y* ^2^ = 1. In fact, substituting the exact solution of Eq. (41), it is straightforward to realize that $X = {\mathrm{tanh}}( \mp (t - t^ \star ))$ and $Y = {\mathrm{sech}}(t - t^ \star )$, resulting in the circular dynamics being a realization of the simple identity $\mathop {{\tanh}}\nolimits^2 + {\mathrm{sech}}^2 = 1$.

### An asymptotically self-similar ODE case

We so far focused on genuinely self-similar examples; the corresponding ideas can also be extended to asymptotically self-similar cases that are not genuinely self-similar in that they possess offending terms, yet upon approaching the singularity the self-similar terms dominate, with the offending ones playing a progressively less important role. Our approach can easily be adapted to this case.

Our simple example variant here will be of the form:

45 $$\dot x = 2x + x^2.$$

Direct integration again can yield the exact solution in the form:

46 $$x(t) = \frac{{2e^{2(t - t^ \star )}}}{{1 - e^{2(t - t^ \star )}}}.$$

It can be seen (when integrating Eq. (45)) that in this case the observable log(*x*/(*x* + 2) is the one that linearly crosses through 0 (as $2(t - t^ \star )$). For *x* large, this quantity becomes

47 $${\mathrm{log}}\left( {\frac{1}{{1 + \frac{2}{x}}}} \right) \approx - 2\frac{1}{x} + 2\frac{1}{{x^2}} - \frac{8}{3}\frac{1}{{x^3}}.$$

Hence, indeed at large times, it is the quadratic term that takes over since the dominant behavior of *x*(*t*) is like $1{\mathrm{/}}(t^ \star - t)$. However, as $t \to t^ \star$, the relevant asymptotics reads:

48 $$x(t) = \frac{1}{{t^ \star - t}} - 1 + \frac{{t^ \star - t}}{3} - \frac{{(t^ \star - t)^3}}{{45}} + \ldots $$

enabling one to observe the explicit (lower order) contribution of the terms offending to the self-similarity. The collapse time, denoted by t*, is still determined by the initial data as $t^ \star = - (1{\mathrm{/}}2){\mathrm{log}}(x(0){\mathrm{/}}(x(0) + 2))$.

Nevertheless, in this case as well, our computational prescription can be carried out. Eq. (45) can be integrated until *x* becomes large. we then revert to *y* = 1/*x* which has the straightforward ODE dynamics:

49 $$\frac{{{\mathrm{d}}y}}{{{\mathrm{d}}t}} = - 2y - 1,$$

(using the transformation to obtain the initial condition *y*(0)) and the equally simple solution *y*(*t*) = −1/2 + (*y*(0) + 1/2)*e* ^−2*t*^. The solution of the latter problem of Eq. (49) crosses 0 en route to its approach of the asymptotic value of −1/2. Finally, once the infinity has been bypassed, we return to the simulation of Eq. (45), as before.

Mapping the dynamics to a circle. The solution of Eq. (45) can be rewritten as:

50 $$x(t) = \frac{2}{{e^{2\left( {t^* - t} \right)} - 1}} \Rightarrow x\frac{{\left( {e^{2\left( {t^* - t} \right)} - 1} \right)}}{2} = 1.$$

Using the compactification the exact same way as Eqs. (3), (4) of the main text and only replacing t* − *t* with: $\left( {e^{2\left( {t^* - t} \right)} - 1} \right){\mathrm{/}}2$, the compactification scheme carries through.

- In this case, if *t* → t*, we Taylor expand and retrieve (from the first term) the limit of exactly Eqs. (3), (4). This is the contribution that stems from the *x* ^2^ term in the ODE.
- In the case of *t* → 0 (or anyway far from t*) the exponential dominates and the (−1) coming from the *x* ^2^ term is irrelevant. This is the contribution that stems from the 2*x* term in the ODE.

### The time between infinities

Following numerous works (including refs. ^26,27^ in the main text), we consider a radial contour along the complex plane i.e., the arc of a circle from the real to the positive imaginary axis. Then, along this arc (denoted by C), we have for *T*, the elapsed time:

51 $$T = {\int}_C {\mathrm{d}}t = {\int}_C \frac{{{\mathrm{d}}z}}{{z^3}} = {\int}_0^{\pi /2} \frac{{Rie^{i\phi }}}{{R^3e^{3i\phi }}}{\mathrm{d}}\phi .$$

Bearing in mind the radial nature of the contour (which renders *R* constant), factoring out 1/*R* ^2^ and taking the limit as *R* → ∞, we obtain a vanishing result, even though the angular integral amounts to unity. That is, interestingly, it takes a finite time to reach from everywhere along the real axis an equidistant point along the imaginary axis, yet this time vanishes as we approach infinity, in line with the analytical result for $\dot x = x^3$. In the case of $\dot z = z^2$, there is a similar result justifying the infinitesimal time of return there from the positive to the negative real axis.

### One and two degree of freedom biologically inspired examples

**Fig. 6:**
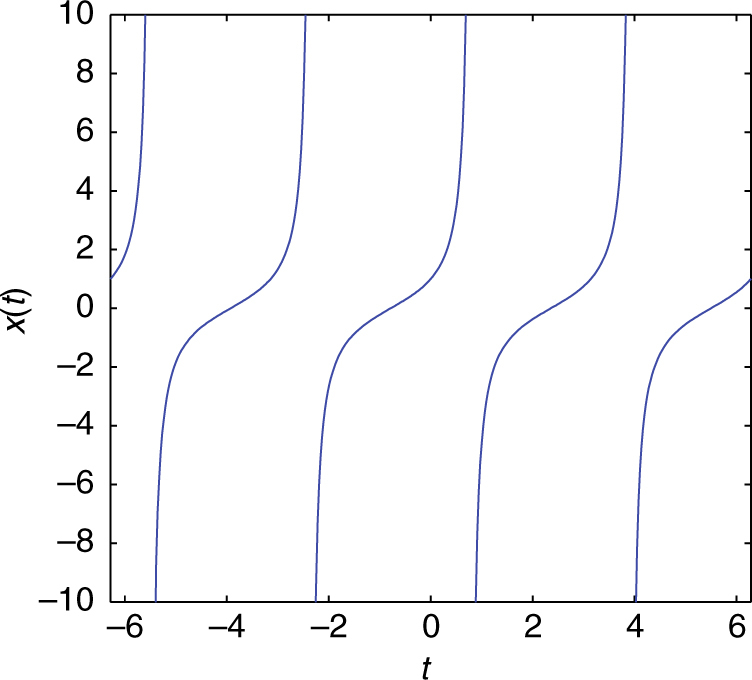
Analytical solution of the 1D quadratic integrate-and-fire neuronal model given by Eq. (52) for *I* = 1

To provide a biologically relevant example of ODE blowup, we start from the following example of ref. ^12^ [Ch. 8, Eq. (8.2)], namely a quadratic integrate-and-fire neuron model. The voltage variable is described here by:

52 $$\frac{{{\mathrm{d}}x}}{{{\mathrm{d}}t}} = I + x^2,$$

where *x* blows up in finite time. The analytical solution of this ODE is $x(t) = \sqrt I \,{\mathrm{tan}}\left( {\sqrt I (c + t)} \right)$. For *I* = 1, *x*(0) = 1 the specific solution is *x*(*t*) = tan(*π*/4 + *t*) (shown in Fig. 6).

While the general transformation (e.g., for *I* = 1) $v = {\int}_x 1{\mathrm{/}}(1 + s^2){\mathrm{d}}s$ can always be used, leading to d*v*/d*t* = −1, it is also possible to be guided by the dominant nonlinear term toward a transformation *y* = 1/*x* which, intriguingly, leads to:

53 $$\frac{{{\mathrm{d}}y}}{{{\mathrm{d}}t}} = - 1 - Iy^2.$$

Although the latter equation has a similar behavior as our original one, possessing blowup features, following our established numerical scheme, namely

**Fig. 7:**
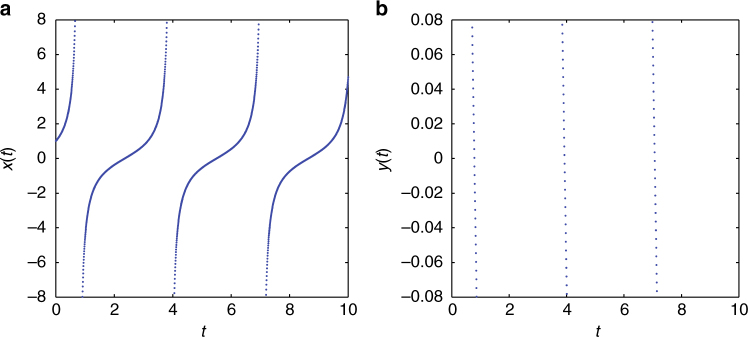
Passing infinity numerically for the 1D quadratic integrate-and-fire neuronal model. Repeatedly: **a** integrate Eq. (52) until *x* sufficiently approaches blowup, **b** integrate the transformed Eq. (53) to pass *y* smoothly through 0. For our simulations, we have set *I* = 1, initial condition *x*(0) = 1; time integration was performed using the ode45 matlab solver^55^ with Relative tolerance = 1.*E* − 08 and absolute tolerance = 1.*E* − 08

1. Integrate Eq. (52) until *x* blows up and then
2. Integrate Eq. (53) to pass *y* smoothly through 0, and finally
3. switch back to Eq. (52) after ∞ has been safely crossed,

will again work as shown in Fig. 7.

It is also sometimes possible to extend our considerations to a setting involving multiple ODEs. In particular, once again inspired by the biologically relevant dynamical systems of ref. ^12^, we select an example of fast-slow dynamics describing the activation of K^+^ and inactivation of Na^+^ current [see Ch. 8, Eqs. (8.3)–(8.4) of ref. ^12^]. More specifically, we have:

54 $$\frac{{{\mathrm{d}}v}}{{{\mathrm{d}}t}} = I + F(v) - u,\quad \frac{{{\mathrm{d}}u}}{{{\mathrm{d}}t}} = bv - u.$$

For the quadratic model corresponding to *F*(*v*) = *v* ^2^, both variables blow up in finite time^14^.

For simulation purposes, to avoid reaching infinity, one clips the voltage in the above models as proposed in ref. ^12^ with a resetting feature at some sufficiently large (cutoff) value. However as discussed in ref. ^40^, the cutoff value has no biophysical interpretation and adds an extra artificial parameter to the model. Importantly, it has been also shown that the system dynamics are very sensitive to changes in the cutoff value, therefore rendering simulation robustness problematic^12,14^. Here, we consider their potential approach to ∞, bearing in mind that^12,14^ suggests that it may be a useful theoretical and computational consideration to set the resetting voltage to ∞.

For this kind of behavior, i.e., when a simultaneous blow up of both variables is observed, we use a polar coordinate decomposition: *v* = *ρ* sin(*θ*) and *u* = *ρ* cos(*θ*), which captures the concurrent blowup of *v* and *u* through that of *ρ*. The resulting equations read (for *b* = 1):

55 $$\begin{array}{l}\frac{{{\mathrm{d}}\rho }}{{{\mathrm{d}}t}} = \rho ^2\,{\mathrm{sin}}^3\theta - \rho \,{\mathrm{cos}}^2\theta + I\,{\mathrm{sin}}\,\theta ,\\ \frac{{{\mathrm{d}}\theta }}{{{\mathrm{d}}t}} = {\mathrm{sgn}}\left( \rho \right)\rho \,{\mathrm{sin}}^2\theta \,{\mathrm{cos}}\theta - 1 + {\mathrm{cos}}\,\theta \,{\mathrm{sin}}\,\theta + \frac{I}{\rho }{\mathrm{cos}}\,\theta .\end{array}$$

**Fig. 8:**
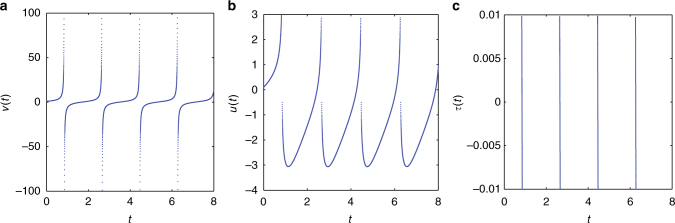
Passing infinity numerically for the 2D quadratic integrate-and-fire neuronal model. Repeatedly: **a**, **b** integrate Eq. (54) until ν sufficiently approaches blowup, **c** integrate the transformed Eq. (55) to pass smoothly through 0. Here we show the dynamics of the singular transformation *r* = 1/*ρ*. For our simulations, we have set *b* = 1, *I* = 1, initial conditions ν(0) = 1, *u*(0) = 0.1; time integration was performed again using the ode23s matlab solver^55^ with Relative tolerance = 1.*E* − 08 and absolute tolerance = 1.*E* − 08

It is now possible to use the singular transformation *r* = 1/*ρ* to once again bypass the finite time singularity of the (*ρ*, *θ*) model by a zero crossing of the (*r*, *θ*) one. Once the singularity is crossed, it is possible to return both to (*ρ*, *θ*), as well as to the original (*u*, *v*) variables. An example of this type is shown in Fig. 8.

**Fig. 9:**
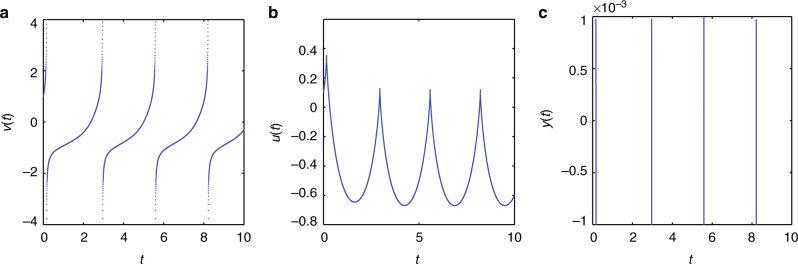
Passing infinity numerically for the 2D quartic integrate-and-fire model. Repeatedly: **a** integrate Eq. (54) until ν, blows up, **b** the adaptation variable *u* remains bounded, **c** integrate the transformed system to pass smoothly through 0. Here we show the dynamics of the singular transformation *y* = 1/ν^3^. For our simulations, we have set *b* = 1, *I* = 1, initial conditions ν(0) = 1, *u*(0) = 0.1; time integration was performed using the ode23s matlab solver^55^ with relative tolerance  = 1.*E* − 08 and absolute tolerance = 1.*E* − 08

Finally, we close this section by offering two remarks: It is straightforwardly possible to envision variants of Eq. (54) which would involve a single degree of freedom blowing up; such an example may be obtained by replacing the nonlinearity *F*(*v*) in the first of Eq. (54) by a quartic one: *F*(*v*) = *v* ^4^ + 2*v*. Such a case is structurally simpler than that where both variables blow up simultaneously. Here as shown^14^ the adaptation variable, *u* remains bounded when *v* blows up. Hence, we need to perform a single transformation such as *y* = 1/*v* ^3^ and consider the system in the transformed (zero crossing in *y*) variables (*y*, *u*). Following the proposed numerical protocol, ∞ is then smoothly crossed (Fig. 9).

The second remark is that similar examples to the polar coordinate one discussed above have been considered (results not shown) in our studies in completely different systems, such as the one stemming from complex forces and complex orbits. A relevant system is that of Eq. (19) of the Contopoulos and Bozis model^41^ which slightly transformed reads: d*v*/d*t* = 2*vu* and d*u*/d*t* = 2*v* ^2^. Here too, the idea of a polar decomposition can be used to capture the concurrent infinity crossing of the two variables.

In summary, the techniques presented herein enable a consideration of settings for ODEs in *R* ^*n*^ where two (or more) degrees of freedom blow up simultaneously or only some of them do.

### Future challenges

Naturally, there are numerous directions of interest for potential future studies. Clearly, exploring additional examples and examining whether the ideas can be equally successfully applied to them is of particular relevance. In the context of ODEs, this is especially relevant as regards vector/multidimensional systems. In the same spirit, exploring further the dynamics of complex ODE models both as generalizations of real ones, but also in their own right, promises to yield useful insights on blowup phenomena (and how to possibly avoid them)^42^; see also the lectures of ref. ^43^ on complex variable dynamics. A related, especially important part in the realm of ODEs is that of convergence of algorithms e.g., to fixed points (or extremizers) of functions. Recall that in such cases, a concern always is whether the code may diverge along the way, rather than reach a root (or an extremum). Our approach can be used to devise algorithms with the ability to systematically bypass infinities during the algorithmic iterations and such a boosted algorithm may be useful toward achieving enhanced, possibly convergence to the roots (or extrema) of a function. This is particularly interesting now that continuous time versions of time-honored discrete algorithms like Newton or Nesterov iteration schemes have become a research focus; see the related discussions in refs. ^44–46^.

On the PDE side, we are envisioning (and currently starting to explore) a multitude of emerging aspects. For instance, when a distributed waveform reaches infinity at a single point in space-time, different post-collapse outcomes are possible. For example, an alternative possibility to the infinity-crossing presented here has been argued to be that the solution may depart from infinity without crossing (the transient blowup in ref. ^29^), as in the case of the standard collapsing NLS equation discussed extensively in textbooks^5,6^. There, the crossing through infinity is precluded by the existence of conservation laws. Past the initial point, it is argued in refs. ^6,21^ that the solution will return from infinity incurring a loss of phase. At the bifurcation level, the work of ref. ^27^ offers a suggestion of how the return from infinity manifests itself: there, a solution with a positive growth rate was identified, that was dynamically approached during the collapse stage. Yet a partial mirror image of that, with negative growth rate, which presumably is followed past the collapse point in order to return from infinity was also identified; see, in particular, Fig. 1 and especially Fig. 2 of ref. ^27^.

It is also possible that such a touch and return from infinity may occur without the loss of phase as, e.g., in the recent work of ref. ^47^. In examining a nonlocal variant of NLS (motivated by ${\cal P}T$-symmetric considerations, i.e., systems invariant under the action of parity and time-reversal), ref. ^47^ identified a solution that goes to infinity in finite time that can be theoretically calculated; subsequently this solution returns from infinity and then revisits infinity again, in a periodic way, always solely touching it and never crossing. This solution is analytically available in Eq. (22) of ref. ^47^ and the collapse times are given by Eq. (23) therein; perhaps even more remarkably, the model itself is integrable. In this case, infinity is reached, subsequently returned from and then periodically revisited. Such an observation would arise in our context if the original PDE for *u* (i.e., a variant of Eq. (12)) had a spatiotemporal limit cycle that attained somewhere in space an extremal value *r*. Then, *w*(*x*, *t*) ≡ 1/(*u*(*x*, *t*) − *r*) would feature the above phenomenology. Such cases where infinity is reached but not crossed merit separate examination. The same is true for solutions exhibiting entire intervals at infinity, whose support progressively grows (or anyway remains finite), bordered by moving fronts; here one may envision that the good equation develops compacton-like solutions^30^.

A related issue that may be worth exploring with such techniques is the possibility of bursting mechanisms (e.g., refs. ^48,49^ involving heteroclinic connections with entire invariant planes at infinity) and the associated emergence of extreme events in nonlinear PDEs. Generalizations of the techniques developed herein to settings where, rather than *u*(*x*, *t*), *u*_*x*_(*x*, *t*) → ∞ (or this happens for other quantities associated with the dependent variable), as is, e.g., the case during the formation of shocks, should also be interesting to explore. Effectively, our considerations here can be thought of as identifying and numerically evolving the infinity level set of the solution. Thus, a related interesting direction for future work could be to try to connect the considerations herein with ones of level set methods^50,51^, adapting the latter toward capturing, e.g., the regions of the singular buffers.

Equally relevant are explicit examples similar to the one herein where multiple collapses may occur and propagate. An intriguing such case is the defocusing scenario of the nonlinear Schrödinger equation,

56 $$iu_t = u_{xx} - 2\left| u \right|^2u,$$

which, in fact, has been shown in ref. ^52^ to possess solutions such as *u*(*x*, *t*) = 1/*x*, or

57 $$u(x,t) = \frac{{2x(x^2 + 6it)}}{{x^4 - 12t^2}},$$

with propagating singularities at *x* = ±12^1/4^ *t* ^1/2^, and

58 $$u(x,t) = \frac{{3\left( {x^8 + 16itx^6 - 120t^2x^4 + 720t^4} \right)}}{{x\left( {x^8 - 72t^2x^4 - 2160t^4} \right)}}.$$

It is obvious that to follow such dynamical examples, a methodology bearing features such as the ones discussed above is needed in order to bypass the continuously propagating singular points. In turn, generalizing such notions to higher dimensions (e.g., a two-dimensional variant of the analytically tractable example herein) and addressing collapsing waveforms both at points, as well as in more complex geometric examples such as curves^53^ is of particular interest for future studies. It is tempting to explore whether the tools developed here may have something to add in the way we analyze collapse in well-established PDEs like the Navier−Stokes, or even singularities arising in a cosmological context. In the context of, e.g., the Navier−Stokes equation, it may be possible to apply relevant transformations either to the original equations for the velocity or, per fundamental results such as the Beale−Kato−Majda criterion^54^, in those for the vorticity. Several of these topics are under active consideration and we hope we will be able to report on them in future publications.

### Data availability

The data that support the findings of this study are available from the corresponding author upon reasonable request.
